# Psychometric Validation of the Arabic Fear of Illness and Virus Evaluation

**DOI:** 10.3390/ijerph18168529

**Published:** 2021-08-12

**Authors:** Abrar Tounsi, Shahad Alammar, Nassr Almaflehi, Mohamed Bamashmous, Abdullah Al Madani, Maria Salem Ibrahim

**Affiliations:** 1 Department of Periodontics and Community Dentistry, College of Dentistry, King Saud University, Riyadh 11451, Saudi Arabia; nassr@ksu.edu.sa; 2 College of Dentistry, Imam Abdulrahman Bin Faisal University, Dammam 34212, Saudi Arabia; alammarshahad@gmail.com; 3 Department of Dental Public Health, Faculty of Dentistry, King Abdulaziz University, Jeddah 21589, Saudi Arabia; Mbamashmous@kau.edu.sa; 4 Dental Hospital, College of Dentistry, Imam Abdulrahman Bin Faisal University, Dammam 34212, Saudi Arabia; aaalmadani@iau.edu.sa; 5 Department of Preventive Dental Sciences, College of Dentistry, Imam Abdulrahman Bin Faisal University, Dammam 34212, Saudi Arabia

**Keywords:** fear, psychometric validation, anxiety, COVID-19, Arabic, FIVE

## Abstract

Global infectious pandemics can affect the psychology and behavior of human beings. Several tools were developed to evaluate the psychological impact of such outbreaks. The present study aimed to examine the psychometric properties of the Arabic translated version of Fear of Illness and Virus Evaluation scale (FIVE). FIVE is a 35-item tool consisting of four subscales that measure Fears about Contamination and Illness, Fears about Social Distancing, Behaviors Related to Illness and Virus Fears and Impact of Illness and Virus Fears. The tool was translated into Arabic by using a forward–backward translation. The online questionnaire contained the following sections: demographics, FIVE, Fear of COVID-19 Scale (FCV-19S) and face validity questions. Non-probability convenient sampling technique was used to recruit participants via a mobile instant messaging application. Reliability, concurrent validity, face validity and factor analysis were examined. The data consisted of 509 adult participants who reside in Saudi Arabia. The internal consistency of the Arabic FIVE subscales was high (0.84–0.91) with strong concurrent validity indicated by positive correlations of FIVE subscales with FCV-19S. Factor analysis suggested slightly different factor structures (Fears of Getting Sick, Fears that Others Get Sick, Fears of the Impact on Social Life and Behaviors Related to Illness and Virus Fears). Our data showed a better fit using the proposed structures. The Arabic version of the FIVE showed robust validity and reliability qualities to assess fear of COVID-19 on Arabic adult population.

## 1. Introduction

The psychology of human beings can be shaped by surrounding events such as global infectious threats [1]. We are vulnerable to struggle from a number of psychological problems during outbreaks such as fear, anxiety, distress and depression. Fear is a normal defensive emotion either innate or acquired [2]. It consists of a sequence of biological steps to prepare humans for a dreadful incident [2]. However, excessive or chronic fear can be pathological leading to anxiety, depression and other psychological diseases or boost pre-existing conditions [3,4,5].

Infectious outbreaks have increased in recent decades due to globalization. Recently, a new family of coronavirus has emerged and caused pandemic concerns. A number of respiratory infection cases due to unclear origin were first noticed in Wuhan, China, by the end of 2019 [6]. As the number of cases increased, China announced in January 2020 that a new strain from coronavirus family is the cause of this illness. It has rapidly spread across China and then to the whole world in a short period of time causing large numbers of morbidity and mortality [7]. The World Health Organization (WHO, Geneva, Switzerland) declared in February 2020 a new name for the widespread disease caused by 2019-nCoV: coronavirus disease (COVID-19). The International Committee on Taxonomy of Viruses has retitled the formerly tentatively named 2019-nCoV as severe acute respiratory syndrome coronavirus-2 (SARS-CoV-2).

According to the World Health Organization (WHO, Geneva, Switzerland), there were more than 177 million confirmed cases of COVID-19 globally with over 3,800,000 death cases by 20 June 2021 [8]. The number varies in numerous countries with highest cases in Americas, Europe and South-East Asia. The coronavirus signifies a global public health issue which is caused by the severe acute respiratory syndrome coronavirus 2 (SARS-CoV-2) [6]. Fever is the most common symptom associated with COVID-19 followed by cough, dyspnea, myalgia, headache, anosmia and diarrhea [9].

Infection control and public health measures work together to limit the spread of this infection by implementing many precautions [10]. The WHO provided infection control guidelines to limit the spread of COVID-19 based on previous knowledge of MERS and SARS management. These measures included enforced social distance by avoiding close contact with other people, frequent hand washing and avoiding interaction with wild animals [11].

Pandemics due to influenza can impose many psychological stressors such as the threat of self-infection or close ones, separation from family and friends due to isolation, change in daily routine, loss of job, school closure and shortage of food or medicine [1,12]. In addition, people were requested to adapt to new social behaviors, such as maintaining physical distance from others, avoid gatherings in groups with their friends, family or work colleagues, and giving up on their usual leisure pursuits [13]. Home confinement due to COVID-19 had a negative effect on daily routine where all physical activities decreased regardless of its intensity, daily sitting time increased, food consumption and meal patterns were more unhealthy during confinement [14]. Caring for the infected and diseased individuals in addition to the threat of death of friends or close ones can be stressful during outbreaks. There are also indirect stress sources such as frequent exposure to media news about the outbreak that could raise uncertainty and distress levels [4,15,16,17]. Among these stressors, there is a concern about the impact of the crisis on public mental health which may lead to various psychological issues such as fear, anxiety, depression and panic disorder [6].

The nature of SARS-CoV-2 and its rapid transmission amid humans raised global tension due to stigmatization, isolation, economic impact and loss [18,19,20]. Therefore, assessing the presence of excessive fear and the severity of psychological effect of pandemics are essential to assist people in coping with mental problems and eliminate its negative consequences. It is also helpful in understanding possible disruptive or defensive reactions such as stigmatization and non-adherence to rules [21]. A number of tools were developed to assess the psychological influence of COVID-19 outbreak [22,23]. Dr. Jill Ehrenreich-May had developed Fear of Illness and Virus Evaluation scale in English (FIVE), a 35-item self-report measure consisting of four subscales; Fears about Contamination and Illness, Fears about Social Distancing, Behaviors Related to Illness and Virus Fears and Impact of Illness and Virus Fears [24]. Before using the scale in different demographic populations, it is preferred to assess the psychometric properties of the scale in the target language [25]. Therefore, the aim of this study was to examine the psychometric properties of the Arabic translated version of FIVE.

## 2. Materials and Methods

The target population was the general Arabic population aged 18 years and older who are able to read and understand written Arabic language. The recommended sample size for validation studies is between 200 and 400 participants [26]. Data was collected from the population residing in Saudi Arabia using an online-based self-report survey. Non-probability convenient sampling technique was used to recruit participants via WhatsApp. Recruitment was stopped when the planned sample size was reached. The study protocol was approved by King Saud University Institutional Review Board (E-20-4792) and the Scientific Research Unit at College of Dentistry at Imam Abdulrahman Bin Faisal University (EA: 202068). Respondents consented to complete the survey.

### 2.1. Measures

The survey was in Arabic language and was composed of Fear of Illness and Virus Evaluation scale-adult version (Ehrenreich-May), Fear of COVID-19 scale (FCV-19S) [20], demographics and a set of questions for face validity testing [27].

#### 2.1.1. Fear of Illness and Virus Evaluation (FIVE; Ehrenreich-May, Unpublished)

FIVE has three versions (adult, child and parent). The adult version consists of four subscales (Fears about Contamination and Illness, Fears about Social Distancing, Behaviors Related to Illness and Virus Fears and Impact of Illness and Virus Fears) with 35 items in total. In the first two subscales, participants were asked to rate the frequency of feeling fear during the last week on a scale of four (1 for “I am not afraid of this at all” and 4 for “I am afraid of this all of the time”). The third subscale includes rating the frequency of doing specific behaviors within the last week on a scale of four (1 for “I have not done this in last week” and 4 for “I did this all the time last week”). The respondents were asked in the last subscale to rate the truthfulness of each statement on a scale of four (1 for “not true for me at all” and 4 for “definitely true”). The first two subscales (items 1–19) are directly related to fears, the third subscale (items 20–33) is related to behaviors, and the fourth one (items 34–35) is related to functionality.

A sum score and a percentage score can be calculated for each subscale where the maximum sum score for Fears about Contamination and Illness subscale is 36, for Fears about Social Distancing subscale is 40, for Behaviors Related to Illness and Virus Fears subscale is 56, and for Impact of Illness and Virus Fears is 8. A higher score for Fears about Contamination and Illness and Fears about Social Distancing subscales indicates higher severity of fear. A higher score for Behaviors Related to Illness and Virus Fears subscale indicates a greater frequency of fear related behaviors. A higher score for Impact of Illness and Virus Fears subscale reflects a greater level of possible impairment.

#### 2.1.2. Fear of COVID-19 Scale (FCV-19S)

To test FIVE concurrent validity, the FCV-19S was added to the questionnaire. It is a unidimensional measure that consists of seven items [20], and it is used to assess the severity of fear towards COVID-19. Participants were asked to describe their opinion on seven items using a 5-point Likert scale ranging from 1 “Strongly disagree” to 5 “Strongly agree”. A total score is calculated by summing all the scores of the seven items with a possible total score ranging between 7 and 35. A higher score indicates a greater level of fear of COVID-19. The scale has shown robust psychometric properties including high internal consistency (α = 0.82) [20]. The scale had good reliability and validity on a sample of Saudi general population [28].

### 2.2. Translation

The research team who are fluent in Arabic and English translated the tool initially into Arabic. An independent linguistic translator reviewed the translation and discussed changes with the research team. Another professional translator, who had not seen the English version of FIVE, back translated the Arabic version into English. The backward translation was then compared to the original FIVE by the research team. The team checked and finalized the items and decided to keep all questions. Piloting was conducted on 15 participants using an online form to get feedback on language and cultural suitability of all the items. Proposed modifications were applied by the research team. The final version of the scale was then administered using an online form on a larger population.

### 2.3. Statistical Analysis

SPSS version 28.0 statistical package software (IBM Corp., Armonk, NY, USA) was used for statistical analyses. Descriptive statistics were used to understand participants’ characteristics including means and standard deviations (SDs) for continuous variables and frequencies with proportions for categorical variables. Reliability was evaluated using Cronbach’s alpha coefficients (α), McDonald’s Omega (ω), inter-item correlations and corrected item-total correlations. Concurrent validity was assessed by running Pearson correlations between each of the FIVE subscales and FCV-19S. A descriptive analysis was also conducted for face validity questions and presented as proportions, means and SDs.

Factor analysis was performed using SPSS AMOS 26 (IBM Corp., Armonk, NY, USA). Since the psychometric properties of the original measure (including factor structure and dimensionality analysis) have not been published yet, the fit of the data to original structures was assessed by confirmatory factor analysis (CFA). When the fit of the data to the original structures was poor, an exploratory factor analysis (EFA) was executed using principal component analysis (PCA) to reveal the structure of latent variables suggested by the data. The assessment was done on various versions of FIVE. As discussed by a previous study [24], items 1–19 are the main questions in FIVE that help in understanding the extent of fear among individuals, more specifically items 1–9 concerned with fear of self or others getting infected. In addition, the developer did not include items 20–33 when scoring the general fear because they only provide additional information about the change in people’s behaviors due to exposure to an illness or virus [24]. Additionally, items 34–35 are related to functionality but not measuring fear itself.

For CFA, maximum likelihood with robustness to non-normality and non-independence of observations (MLR) was used as the method of estimation for all models. The following fit indices with their conventionally accepted cut-off values [29] were used to assess the fit: chi-square/degree of freedom (X^2^/DF) [1.0–5.0] [30], root mean square error of approximation (RMSEA) [<0.08] [31], standardized root mean square residual (SRMR) [<0.08] [32], comparative fit index (CFI) [33] and Tucker–Lewis fit index (TLI) [34] [>0.90].

## 3. Results

### 3.1. Sample Characteristics

The sample included 509 individuals who completed the Arabic questionnaire (Table 1). Overall, more than half of the participants were females (*n* = 308, 60.51%), the majority had a Saudi nationality (*n* = 476, 93.52%), and half of the included sample had Bachelor qualification (*n* = 262, 51.47%). The mean age of participants was 33.88 ± 10.52 years, ranging between 18 and 70. Half of the participants shared their living with spouse and kids (*n* = 255, 50.10%) and 62.28% were married. About 39% of the sample were working in the governmental sector at the time of participation, and 40.47% were working in the medical field.

### 3.2. Mean Sum Scores of FIVE Subscales

The sample mean score for Fears about Contamination and Illness subscale was 19.72 ± 6.46. For Fears about Social Distancing subscale, the mean score was 19.62 ± 7.67, and the mean scores for Behaviors Related to Illness and Virus Fears and Impact of Illness and Virus Fears subscales were 33.16 ± 8.87 and 3.57 ± 1.81, respectively. Fears about Contamination and Illness and Impact of Illness and Virus Fears were significantly higher among females 20.12 ± 6.48, *p* = 0.02 and 3.79 ± 1.91, *p* < 0.001, respectively. Adults above the age of 50 had significantly lower Fears about Contamination and Illness (16.4 ± 7.73), Behaviors Related to Illness and Virus Fears (17.16 ± 8.11) and Impact of Illness and Virus Fears (3.02 ± 1.80) at *p* < 0.05. However, there was no statistically significant difference between medical field workers and non-medical field workers in any of the FIVE subscales.

### 3.3. Reliability

Overall, the Arabic version of FIVE for adults showed adequate internal consistency (Table 2). The overall internal consistency of Fears about Contamination and Illness subscale (items 1–9) was high (α = 0.89, ω = 0.90). The correlations between the items in the subscale showed significant inter-item correlations ranging between 0.22 and 0.79, and corrected item-total correlations ranged between 0.36 and 0.77.

The correlation matrix of the second subscale (Fears about Social Distancing; items 10–19) showed a strong internal consistency (α = 0.91, ω = 0.91) with significant inter-item correlations ranging between 0.31 and 0.75 (*p* < 0.01) and corrected item-total correlations ranging between 0.57 and 0.74.

The internal consistency of the third subscale (Behaviors Related to Illness and Virus Fears; items 20–33) was strong (α = 0.87, ω = 0.87) with significant inter-item correlations ranging between 0.10 (*p* < 0.05) and 0.67 (*p* < 0.01) and corrected item-total correlations ranging between 0.31 and 0.65.

The fourth subscale (Impact of Illness and Virus Fears; items 34 and 35) had a strong internal consistency (α = 0.84) and a significant inter-item correlation of 0.72 (*p* < 0.01).

### 3.4. Concurrent Validity

When the correlation of FIVE subscales was assessed in relation to FCV-19S as shown in Table 2, each of Fears about Contamination and Illness, Fears about Social Distancing, Behaviors Related to Illness and Virus Fears and Impact of Illness and Virus Fears subscales were significantly correlated with FCV-19S (*p* < 0.001). The highest correlation of FCV-19S was noticed with Fears about Contamination and Illness (*r* = 0.54) and Impact of Illness and Virus Fears (*r* = 0.51).

### 3.5. Face Validity

The face validity of the Arabic version of FIVE is presented in Table 3. Most of respondents endorsed the clearness and easiness of the questions (80.73%) and on covering all areas regarding fear of illness and virus (69.95%). More than half (56.98%) of the respondents would like to use the Arabic version of FIVE for their future assessment. A high percentage of the respondents disapproved that the Arabic version of FIVE lacks important questions regarding their fear of illness and virus (55.20%) or violates their privacy (78.59%).

### 3.6. Factor Analysis

The conformity of our data to the original structure was tested using CFA (Table 4). Due to the poor fit, an EFA for items 1–35 was performed to find out an alternative structure (Table 5). The analysis yielded four factors, accounting for 52.70% of the total variance of the data. The four factors were Fears of Getting Sick, Fears that Others Get Sick, Fears of the Impact on Social Life and Behaviors Related to Illness and Virus Fears. Both original Fears about Social Distancing and Impact of Illness and Virus Fears tended to load on the same factor along with item 5 “I am afraid my pet might get a bad illness or virus”. While original Fears about Contamination and Illness tended to load on two separate factors (Fears of Getting Sick; items 1–4 and Fears that Others Get Sick; items 6–9). Behaviors Related to Illness and Virus Fears kept loading on a single factor. The Bartlett’s test of sphericity indicated that the data are sufficiently correlated to perform EFA: χ^2^ (595) = 10,095.19, *p* < 0.001. Sampling adequacy assessed by Kaiser–Meyer-Olkin (KMO) measurements resulted in a mean sampling adequacy of 0.91, suggesting that the factorability of data is good.

**Figure 1:**
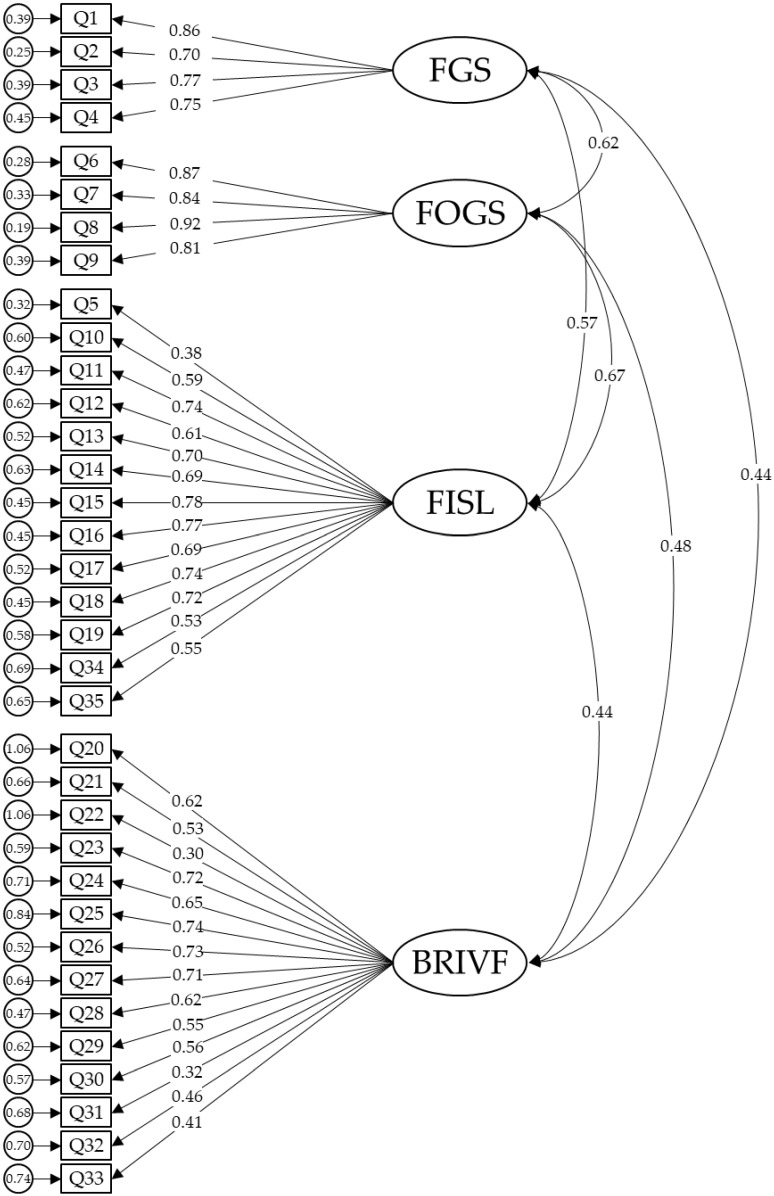
CFA for FIVE proposed four factors. Note: the ellipses represent the factors and the rectangles represent the different items. The residual variances are shown in the small circles. Fears of Getting Sick (FGS), Fears that Others Get Sick (FOGS), Fears of the Impact on Social Life (FISL), Behaviors Related to Illness and Virus Fears (BRIVF).

Fit indices reflected relatively acceptable fit for the proposed four factors model [X^2^/DF = 4.78, TLI = 0.77, CFI = 0.79; RMSEA = 0.08; 90% CI (0.08, 0.09); SRMR = 0.07]. Standardized regression weights, correlation between latent factors and residual variances that were significant at *p* < 0.001 are illustrated for the four factors model in Figure 1. There was a significant improvement in model fit as shown in Table 4. However, it was slightly below the recommended criteria for some indices [29].

Thereafter, the structure of items 1–19 was assessed using EFA (Table 6). It yielded three factors, accounting for 62.35% of the total variance of the nineteen questions. Items of Fears about Contamination and Illness loaded on two separate factors (Fears of Getting Sick; items 1–5 and Fears that Others Get Sick; items 6–9). Items of Fears about Social Distancing (items 10–19) kept loading on a single factor. The Bartlett’s test of sphericity indicated that data are sufficiently correlated to perform EFA: χ^2^ (171) = 6216.87, *p* < 0.001. Sampling adequacy assessed by Kaiser–Meyer-Olkin (KMO) measurements resulted in a mean sampling adequacy of 0.91, suggesting that the factorability of data is good. It is essential to mention that item 5 loaded differently on two latent factors in Table 5 and Table 6 based on the number of items included in the model, but in both cases the factor loading was relatively low yet significant.

**Figure 2:**
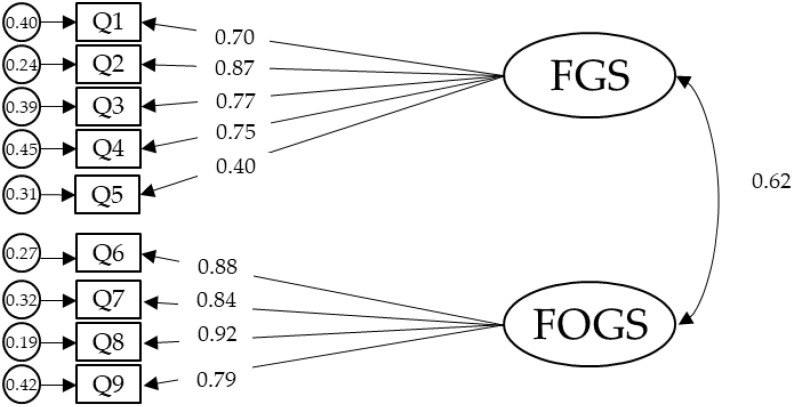
CFA for FIVE proposed two factors. Note: the ellipses represent the factors and the rectangles represent the different items. The residual variances are shown in the small circles. Fears of Getting Sick (FGS), Fears that Others Get Sick (FOGS).

When compared to the original structure of two factors among items 1–19, the fit for the newly proposed three factors model was improved (Table 4) but yet away from the recommended criteria [29]. Therefore, we proceeded with analyzing the structure of items 1–9 (Table 6). The model obtained two factors (Fears of Getting Sick; items 1–5 and Fears that Others Get Sick; items 6–9) accounting for 68.62% of the total variance of the data (Table 6). The proposed two factors model had an acceptable fit [X^2^/DF = 4.68, TLI = 0.95, CFI = 0.97; RMSEA = 0.09; 90% CI (0.07, 0.11); SRMR = 0.03]. Standardized regression weights, correlation between latent factors and residual variances that were significant at *p* < 0.001 are illustrated in Figure 2.

## 4. Discussion

The deleterious impact of infectious pandemics on individuals’ mental health is well documented in the literature [35,36]. Assessing psychological influence of health disasters using various validated measuring tools [20,37,38] facilitates understanding how people would encounter and endure global pandemics and facilitates detecting individuals with mental health needs. Therefore, Fear of Illness and Virus Evaluation (FIVE) is a tool that was developed by *Ehrenreich-May* (*In preparation*) to estimate the severity and the impact of fears of the public during the COVID-19 global outbreak. This tool was translated to Arabic in the present study and reliability qualities, concurrent validity, face validity, exploratory factor analysis and confirmatory factor analysis were evaluated. The overall findings indicated that the Arabic translated version of FIVE for adults is a reliable and valid tool for assessing the severity and the impact of COVID-19 fear among the population of Saudi Arabia.

FIVE consists of 35 items divided into four subscales: Fears about Contamination and Illness, Fears about Social Distancing, Behaviors Related to Illness and Virus Fears and Impact of Illness and Virus Fears. It is a distinguishable tool in being multidimensional and can be useful for multiple purposes as measuring not only fears but behaviors related to fear during the recent pandemic or any other future illnesses.

The conformity between subscales’ items in the current Arabic version of FIVE examined in the present study is comparable to the Arabic Fear of COVID-19 Scale (FCV-19S) [28]. For instance, the internal consistency of Fears about Contamination and Illness subscale (items 1–9) was 0.89 and Fears about Social Distancing (items 10–19) was 0.91, which are relatively higher than that found in the Arabic FCV-19S [28] and Spanish FIVE [24]. The internal consistency of proposed structure of FIVE in Spanish language was α = 0.88 for Fears of Getting Sick from an illness or virus (items 1–4); α = 0.74 for Fears that Others May Get Sick from an illness or virus (items 5–9); α = 0.85 for Fears of Concrete Limitations due to an illness or virus (items 10, 11, 13, 15–17); while the subscale Fears of not being able to meet Basic Needs of subsistence and work due to an illness or virus (items 12, 14, 18, 19) had α = 0.79. The other FIVE subscales were marginally lower than FCV-19S (Behaviors Related to Illness and Virus Fears α = 0.87 and Impact of Illness and Virus Fears α = 0.84). Nevertheless, Cronbach’s alpha of 0.70 or higher is considered as an acceptable reliability [39].

The inter-item correlation analysis of the presented tool generally revealed a satisfactory internal consistency. The inter-item correlations and corrected item-total correlations between 0.30 and 0.70 suggest medium to strong associations between the items [40,41]. However, item 5 “I am afraid my pet might get a bad illness or virus” in Fears about Contamination and Illness subscale had a corrected item-total correlation of *r* = 0.36. This relatively lower correlation reflects its incompatibility in measuring the same attribute of this subscale (Fears about Contamination and Illness) [41]. This finding was further supported by item loading in exploratory factor analysis in which item 5 loaded with Fears of the Impact on Social Life when items 1–35 were included and with Fears of Getting Sick when items 1–19 were included in the analysis. The culture of adopting pets in Saudi Arabia is not widely popular. Therefore, the inclusion of this question in future investigations should be considered based on researchers’ objectives.

Concurrent validity of the present tool indicates positive correlations of FIVE subscales with the Arabic FCV-19S. These findings ascertain that FIVE is credible in assessing the psychological conditions arising from COVID-19. Similarly, FIVE was positively correlated with Hospital Anxiety and Depression Scale [38]. The correlation coefficient of FIVE with Anxiety subscale was high (*r* = 0.83) and moderate with depression subscale (*r* = 0.66) in a previous study [38]. FIVE was also correlated with both depressive symptomatology (PHQ-9) and posttraumatic stress (ITQ) [24].

Participants ascertained the clearness and easiness of the survey questions and the comprehensiveness of the questionnaire to important aspects of fear from COVID-19. Most of the respondents denied that the questionnaire violated their privacy. However, few felt the opposite, which is in line with the Hospital Anxiety and Depression Scale where authors reflected the possible sensitivity of some items involved in psychological assessment [27,40]. This would emphasize the importance of being more sensible during psychological evaluations.

The suggested structure for FIVE in the present study is based on its factor analysis and model fit indices. As a previous study [24] suggested four domains for items 1–19 (Fears of Getting Sick, Fears that Others May Get Sick, Fears of Concrete Limitations and Fears of not being able to meet the Basic Needs), our findings that were based on a sufficiently larger and sociodemographically different sample obtained a structure (Fears of Getting Sick; items 1–5, Fears that Others Get Sick; items 6–9 and Fears of the Impact on Social Life; items 10–19) that is somewhat different from that proposed in the Spanish version. On the other hand, when items 1–35 were analyzed, the data showed an improved fit in four domains that was slightly different from the one originally set by *Ehrenreich-May.* The newly proposed structure included Fears of Getting Sick; items 1–4, Fears that Others Get Sick; items 6–9, Fears of the Impact on Social Life; items 5, 10–19, 34–35 and Behaviors Related to Illness and Virus Fears; items 20–33. The proposed structure of two factors (items 1–9) had the best fit among other structures. Therefore, future intentions to use this tool in its current Arabic format are recommended to select one of the newly proposed structures that best serves their purposes.

The sample used in the present study comprised of 60% females, over 50% Bachelor qualification or higher and more than 90% were Saudi nationals. The mean age of participants was 33.88 years. Other demographic characteristics might have different findings in different populations. Upcoming studies must inspect whether the current Arabic FIVE version attains comparable psychometric properties in larger and randomly selected samples. Sociodemographic factors, such as age and gender, play a role in the perception of fears from illness and viruses; thus, it is recommended to run multi-group invariance tests in the future to examine the differences in perception of FIVE items across different age groups, genders and other sociodemographic characteristics.

The fear of COVID-19 has a positive relationship with anxiety and depression [19,20,38]. In some extreme cases, fear of COVID-19 was associated with suicide attempts [42,43]. Social isolation, economic impact, continuous exposure to the intimidating news about the pandemic and uncertainty may increase the level of anxiety, fear and depression [20,44]. In addition, the doubts about contracting SARS-CoV-2 could increase fear amongst people [18]. Therefore, it is advisable to implement targeted prevention and education programs to aid individuals who are or at risk of being overwhelmed by fear from COVID-19 and help such individuals to engage in preventative behaviors [20,45].

Although the present study demonstrated that the Arabic version of FIVE is a reliable and valid tool for estimating the severity of fear from COVID-19, it has some limitations. First, the studied participants were from the general population in Saudi Arabia without professional psychological assessment. Consequently, the sensitivity and specificity of FIVE subscales could not be inspected. In addition, the dependence on an online survey and self-report measurement is influenced by social desirability which could underestimate the severity of fear [40]. Moreover, a convenient sample was used for this study which may affect the generalizability of our findings. The lack of invariance measurement could be another limitation of this paper.

In summary, based on the sampled population, the present study indicates that the adopted Arabic version of FIVE has good internal consistency and reliability. We consider it a suitable tool that can be used in research to assess the severity of fear of COVID-19 or any other virus on Arabic populations.

## Figures and Tables

**Table 1:** Sample characteristics.

Age (Years)	Minimum	18
	Maximum	70
	Mean ± SD	33.88 ± 10.52
	Under 25	128 (25.15)
	Between 25 and 50	334 (65.62)
	Above 50	47 (9.23)
Gender *n* (%)	Male	201 (39.49)
	Female	308 (60.51)
Nationality *n* (%)	Saudi	476 (93.52)
	Non-Saudi	33 (6.48)
Educational Level *n* (%)	High school or less	74 (14.54)
	Diploma	54 (10.61)
	Bachelor	262 (51.47)
	Postgraduate	119 (23.38)
Living Status *n* (%)	Spouse	31 (6.09)
	Spouse and kids	255 (50.10)
	Parents	118 (23.18)
	Alone with/without kids	25 (4.91)
	Other family members	60 (11.79)
	Other	20 (3.93)
Marital Status *n* (%)	Single	180 (35.36)
	Married	317 (62.28)
	Divorced or widow	12 (2.36)
Employment Status *n* (%)	Student	135 (26.52)
	Unemployed	46 (9.04)
	Private sector	88 (17.29)
	Governmental sector	199 (39.10)
	Retired	41 (8.05)
Works in the Medical Field *n* (%)	Yes	206 (40.47)
	No	303 (59.53)

**Table 2:** Distribution, internal reliability and correlation of Fear of Illness and Virus Evaluation Subscales.

		Fears about Contamination and Illness	Fears about Social Distancing	Behaviors Related to Illness and Virus Fears	Impact of Illness and Virus Fears
Distribution	Skewness	0.28	0.87	0.25	1.04
	Kurtosis	−0.60	0.02	−0.16	0.06
Internal Reliability	Cronbach’s alpha	0.89	0.91	0.87	0.84
	McDonald’s Omega	0.90	0.91	0.87	-
Correlations	Fears about Contamination and Illness	1			
	Fears about Social Distancing	0.64 **	1		
	Behaviors Related to Illness and Virus Fears	0.48 **	0.42 **	1	
	Impact of Illness and Virus Fears	0.44 **	0.51 **	0.33 **	1
	FCV-19S	0.54 **	0.44 **	0.47 **	0.51 **

** Correlation is significant at the 0.001 level (2-tailed).

**Table 3:** Descriptive statistics for FIVE face validity.

	Strongly Disagree *n* (%)	Disagree *n* (%)	Neutral *n* (%)	Agree *n* (%)	Strongly Agree *n* (%)	Mean ± SD
1. Questions were clear and easy	17 (3.34)	30 (5.89)	51 (10.02)	291 (57.17)	120 (23.58)	3.92 ± 0.93
2. Questions covered all my problem areas with fear of illness and virus	14 (2.75)	44 (8.64)	95 (18.66)	269 (52.85)	87 (17.10)	3.73 ± 0.94
3. I would like the use of this questionnaire for future assessments	23 (4.52)	61 (11.98)	135 (26.52)	224 (44.01)	66 (12.97)	3.49 ± 1.01
4. The questionnaire lacks important questions regarding my fear of illness and virus	125 (24.55)	156 (30.65)	113 (22.20)	87 (17.10)	28 (5.50)	2.48 ± 1.19
5. Some of the questions violate my privacy	272 (53.44)	128 (25.15)	42 (8.25)	47 (9.23)	20 (3.93)	1.85 ± 1.15

**Table 4:** Model fit indices for CFA.

	X^2^/DF	TLI	CFI	RMSEA (90% CI)	SRMR
Original structure					
Four factors (items 1–35)	5.26	0.74	0.76	0.09 (0.09, 0.10)	0.08
Two factors (items 1–19)	10.38	0.74	0.77	0.14 (0.13, 0.14)	0.08
Proposed new structures					
Four factors (items 1–35)	4.78	0.77	0.79	0.08 (0.08, 0.09)	0.07
Three factors (items 1–19)	6.96	0.83	0.85	0.11 (0.10, 0.12)	0.07
Two factors (items 1–9)	4.68	0.95	0.97	0.09 (0.07, 0.11)	0.03

Note: chi square (X^2^), degree of freedom (DF), Tucker–Lewis fit index (TLI), comparative fit index (CFI), root mean square error of approximation (RMSEA) with its confidence interval (CI) at 90%, and standardized root mean square residual (SRMR).

**Table 5:** Factor analysis for FIVE items 1–35.

	Component			
Item Number	Fears of Getting Sick	Fears That Others Get Sick	Fears of the Impact on Social Life	Behaviors Related to Illness and Virus Fears
1	0.68			
2	0.67			
3	0.64			
4	0.61			
5			0.40	
6		0.51		
7		0.55		
8		0.57		
9		0.43		
10			0.63	
11			0.74	
12			0.63	
13			0.70	
14			0.71	
15			0.79	
16			0.77	
17			0.72	
18			0.74	
19			0.75	
20				0.64
21				0.48
22				0.32
23				0.73
24				0.67
25				0.75
26				0.71
27				0.71
28				0.67
29				0.54
30				0.59
31				0.37
32				0.44
33				0.45
34			0.53	
35			0.57	

Extraction method: principal component analysis. Rotation method: Quartimax with Kaiser normalization.

**Table 6:** Factor analysis for FIVE items 1–19 and 1–9.

Item Number	Component		
	Fears of the Impact on Social Life	Fears of Getting Sick	Fears That Others Get Sick
1		0.75 (0.71)	
2		0.81 (0.82)	
3		0.79 (0.80)	
4		0.76 (0.80)	
5		0.41 (0.50)	
6			0.80 (0.84)
7			0.81 (0.86)
8			0.84 (0.90)
9			0.73 (0.83)
10	0.66		
11	0.71		
12	0.59		
13	0.73		
14	0.63		
15	0.73		
16	0.71		
17	0.75		
18	0.71		
19	0.65		

Extraction method: principal component analysis. Rotation method: Varimax with Kaiser normalization. Numbers inside the parentheses represent factor loadings for two factors model (items 1–9).

## Data Availability

The data presented in this study are available on request from the corresponding authors. The data are not publicly available due to privacy restrictions.
